# Critical Amino Acid Variants in HLA-DRB1 and -DQB1 Allotypes in the Development of Classical Type 1 Diabetes and Latent Autoimmune Diabetes in Adults in the Japanese Population

**DOI:** 10.3390/cimb43010009

**Published:** 2021-05-09

**Authors:** Masahito Katahira, Taku Tsunekawa, Akira Mizoguchi, Mariko Yamaguchi, Kahori Tsuru, Hiromi Takashima, Ryoma Terada

**Affiliations:** 1 Nursing and Health, Aichi Prefectural University School of Nursing and Health, Togoku, Kamishidami, Moriyama-ku, Nagoya 463-8502, Japan; 2 Department of Endocrinology and Diabetes, Ichinomiya Municipal Hospital, 2-2-22, Bunkyo, Ichinomiya 491-8558, Japan; tsune-ta@med.nagoya-u.ac.jp (T.T.); west2coast12@yahoo.co.jp (A.M.); mari-y@med.nagoya-u.ac.jp (M.Y.); ra28537@qf6.so-net.ne.jp (K.T.); g.a.crown23@gmail.com (H.T.); teraterar@gmail.com (R.T.)

**Keywords:** HLA class II, type 1 diabetes, latent autoimmune diabetes in adults, amino acid variants

## Abstract

The effects of amino acid variants encoded by the human leukocyte antigen (HLA) class II on the development of classical type 1 diabetes (T1D) and latent autoimmune diabetes in adults (LADA) have not been fully elucidated. We retrospectively investigated the *HLA-DRB1* and *-DQB1* genes of 72 patients with classical T1D and 102 patients with LADA in the Japanese population and compared the frequencies of *HLA-DRB1* and *-DQB1* alleles between these patients and the Japanese populations previously reported by another institution. We also performed a blind association analysis with all amino acid positions in classical T1D and LADA, and compared the associations of HLA-DRB1 and -DQB1 amino acid positions in classical T1D and LADA. The frequency of DRß-Phe-13 was significantly higher and those of DRß-Arg-13 and DQß-Gly-70 were significantly lower in patients with classical T1D and LADA than in controls. The frequencies of DRß-His-13 and DQß-Glu-70 were significantly higher in classical T1D patients than in controls. The frequency of DRß-Ser-13 was significantly lower and that of DQß-Arg-70 was significantly higher in LADA patients than in controls. HLA-DRß1 position 13 and HLA-DQß1 position 70 could be critical amino acid positions in the development of classical T1D and LADA.

## 1. Introduction

Type 1 diabetes (T1D) has three prevalent subtypes: acute-onset (classical), slow-onset, and fulminant T1D [1]. Slow-onset T1D, which develops after the age of 30, is commonly referred to as latent autoimmune diabetes in adults (LADA) [2,3]. Although LADA and fulminant T1D, as well as classical T1D, are associated with human leukocyte antigen (HLA) class II genes, the HLA class II genes that affect the onset of T1D depend on T1D subtype and ethnic group [4,5,6]. In Caucasian populations, HLA-DRB1*03-DQB1*02:01 and -DRB1*04-DQB1*03:02 haplotypes are associated with the highest risk of classical T1D [6] and LADA [4]. Moreover, the existence of DQα-Arg-52 and the absence of DQβ-Asp-57 confer susceptibility to classical T1D [7,8]. Because HLA-DRB1*03-DQB1*02:01 and -DRB1*04-DQB1*03:02 haplotypes are rarely observed in Japanese populations [2,9], it is necessary to investigate T1D susceptibility conferred by HLA class II genes other than HLA-DRB1*03-DQB1*02:01 and -DRB1*04-DQB1*03:02 haplotypes.

We previously demonstrated that HLA-DRB1*04:05-DQB1*04:01, -DRB1*08:02-DQB1*03:02, -DRB1*09:01-DQB1*03:03, and -DRB1*13:02-DQB1*06:04 haplotypes confer susceptibility to classical T1D, and HLA-DRB1*08:02-DQB1*03:02 and -DRB1*09:01-DQB1*03:03 haplotypes confer susceptibility to slow-onset T1D in the Japanese population [2]. However, the role of critical amino acid variants in HLA-DRB1 and -DQB1 alleles in the onset of LADA, such as DQα-Arg-52 and DQβ-Asp-57 in classical T1D, are not fully elucidated. We investigated the HLA-DRB1 and -DQB1 genes in Japanese classical T1D and LADA patients by comparing their allele frequencies with those of controls who were representative of the healthy Japanese population. We also investigated the effects of polymorphic amino acid residue variations in HLA-DRB1 and -DQB1 alleles on the onset of classical T1D and LADA.

## 2. Materials and Methods

### 2.1. Study Participants

This is a retrospective study. We identified 185 unrelated Japanese patients with classical (*n* = 72) and slow-onset (*n* = 113) T1D who visited the internal medicine departments of participating hospitals between 2001 and 2010. The participating hospitals were Ichinomiya Municipal Hospital (Ichinomiya, Japan), Kyoritsu General Hospital (Nagoya, Japan), and Okazaki City Hospital (Okazaki, Japan), all in the Aichi Prefecture. All patients fulfilled the World Health Organization criteria for diabetes [10]; we have reported some of their clinical and immunogenetic characteristics [2,11]. We excluded 11 patients with slow-onset T1D whose onset of diabetes occurred at age <30 years. Thus, 72 unrelated patients with classical T1D and 102 unrelated patients with slow-onset T1D comprised the final classical T1D and LADA cohort.

These frequencies of *HLA-DRB1* and *-DQB1* alleles were compared with those of other Japanese populations, as reported by the Japanese Society of Histocompatibility and Immunogenetics [2,9]. This study was approved by the Ethics Committee of Aichi Prefectural University (approval code: 1-43, 26 February 2021) and was conducted in accordance with the Declaration of Helsinki.

### 2.2. Measurements

The titers of glutamic acid decarboxylase (GAD) antibody (GAD-Ab) and insulinoma-associated antigen-2 antibody (IA-2Ab) were determined as described previously [12]. The cutoff values for GAD-Ab and IA-2Ab were 1.5 and 0.4 U/mL, respectively. Urinary and serum C-peptide levels were determined using a commercially available enzyme immunoassay kit (Eiken C-Peptide Kit; Eiken Chemical, Tokyo, Japan).

*HLA-DRB1* sequence-based typing (SBT) was performed by directly sequencing DRB1 exon 2 using the AlleleSEQR DRB1 Typing Kit (Atria Genetics, San Francisco, CA, USA) according to the manufacturer’s instructions, as described previously [2]. *HLA-DQB1* SBT was carried out by direct sequencing of DQB1 exon 2 and 3 using the AlleleSEQR DQB1 Typing Kit (Atria Genetics) according to the manufacturer’s instructions, as described previously [2]. Ambiguous genotyping samples were identified using high-resolution polymerase chain reaction-sequence-specific amplification and heterozygous ambiguity resolution primer’s methods. The BIGDAWG software, version 2.5, implemented as the *bigdawg* R package (GitHub, San Francisco, CA, USA) [13], was used for the analysis of amino acids encoded by the *HLA-DRB1* and *-DQB1* alleles.

### 2.3. Statistical Analysis

Study results are presented as means ± standard deviation or percentages with numbers. SPSS 26.0 software (IBM Corp., Armonk, NY, USA) was used for statistical analyses. Chi-square tests based on 2 × 2 contingency tables and Fisher’s exact probability tests were used to compare allele frequencies. *p* values were corrected for the number of different alleles tested using the Benjamini–Hochberg method (denoted as *Pc*) [14]. *Pc* < 0.05 was considered statistically significant.

## 3. Results and Discussion

### 3.1. Clinical Data

The median age of patients with classical T1D (29 men and 43 women) was 30 years (range 5–81) at the onset of diabetes. The duration of diabetes and body mass index (BMI) were 9.8 ± 9.9 years and 20.6 ± 3.3 kg/m^2^, respectively. The positive GAD-Ab and IA-2Ab rates were 60.9% and 42.9%, respectively; 21 classical T1D patients (29.2%) had no positive results for GAD-Ab or IA-2Ab. Urinary and serum 2-h C-peptide levels were 2.6 ± 3.5 nmol/day and 0.15 ± 0.24 nmol/L, respectively. All classical T1D patients were treated with insulin and insulin dosage was 0.69 ± 0.30 U/kg/day.

The median age of patients with LADA (55 men and 47 women) was 52 years (range 31–80) at the onset of diabetes. The duration of diabetes and BMI were 9.5 ± 8.7 years and 23.1 ± 4.3 kg/m^2^, respectively. The positive GAD-Ab rate was 100%. Urinary and serum 2-h C-peptide levels were 10.8 ± 12.8 nmol/day and 0.92 ± 0.96 nmol/L, respectively. The rate of LADA patients treated with insulin was 69.6% and insulin dosage was 0.50 ± 0.25 U/kg/day.

### 3.2. HLA-DRB1 and -DQB1 Allele Frequencies in Patients with Classical T1D and LADA

We identified 18 *HLA-DRB1* alleles and 11 HLA-DQB1 alleles in patients with classical T1D and 23 *HLA-DRB1* alleles and 12 *HLA-DQB1* alleles in patients with LADA. The *HLA-DRB1* and *-DQB1* allele frequencies in patients with classical T1D, those with LADA, and controls are presented in Table 1. However, *HLA-DRB1* and *-DQB1* alleles with less than a total of five frequencies in patients with classical T1D, those with LADA, and controls were excluded from the comparison of frequencies.

We previously demonstrated that HLA-DRB1*04:05-DQB1*04:01, -DRB1*08:02-DQB1*03:02, -DRB1*09:01-DQB1*03:03, and -DRB1*13:02-DQB1*06:04 haplotypes confer susceptibility to classical T1D in the Japanese population [2]. Of these alleles, only HLA-DQB1*03:02 allele was not associated with classical T1D in the present study. In Caucasian populations, the HLA-QB1*03:02 allele constitutes the HLA-DRB1*04-DQA1*03:01-DQB1*0302 haplotype, which confers susceptibility to classical T1D [5]. In the Japanese population, this haplotype is rare, but the HLA-DQB1*03:02 allele constitutes the haplotype with HLA-DRB1*08:02 and -DQA1*03:01 alleles, which has Arg at HLA DQα1 position 52 that confers susceptibility to classical T1D [8]. The *HLA-DQA1* allele, rather than the *HLA-DRB1* and *-DQB1* alleles, might play an important role in the development of classical T1D in this haplotype. HLA-DRB1*11:01, -DRB1*14:05, -DRB1*15:01, -DRB1*15:02, -DQB1*03:01, -DQB1*05:03, -DQB1*06:01, and -DQB1*06:02 alleles were found to confer protection against classical T1D in this study. These alleles constitute *HLA-DRB1-DQB1* haplotypes in the Japanese population, i.e., HLA-DRB1*11:01-DQB1*03:01, -DRB1*14:05-DQB1*05:03, -DRB1*15:01-DQB1*06:02, and -DRB1*15:02-DQB1*06:01 [2,9]. The above findings are consistent with previous studies [2,6].

We previously demonstrated that HLA-DRB1*04:07-DQB1*03:02, -DRB1*08:02-DQB1*03:02, and -DRB1*09:01-DQB1*03:03 haplotypes confer susceptibility to slow-onset T1D in the Japanese population [2,11]. Of these alleles, we found that only the HLA-DRB1*08:02 allele was not associated with LADA. The HLA-DRB1*08:02-DQB1*03:02 haplotype shares the same HLA-DQB1 allele with the HLA-DRB1*04:07-DQB1*03:02 haplotype. The HLA-DQB1*03:02 allele might play an important role in the development of LADA in these haplotypes. We found that the HLA-DRB1*15:01 and -DQB1*06:02 alleles confer protection against LADA. These alleles constitute the *HLA-DRB1-DQB1* haplotype [2,9] and the above findings are consistent with previous studies [2,11].

### 3.3. Polymorphic DRB1 and DQB1 Amino Acid Residue Variations in Patients with Classical T1D and LADA

To elucidate why *HLA-DRB1* and *-DQB1* alleles differentially contribute to the development of classical T1D and LADA, we examined the amino acid variants in HLA-DRB1 and -DQB1 allotypes. We performed a blind association analysis of all amino acid positions in classical T1D and LADA using Bridging ImmunoGenomic Data-Analysis Workflow Gaps (BIGDAWG) software and compared the associations of different amino acid positions in classical T1D and LADA. Since the cohort of this study was not very large and belonged to the same ethnic group, we did not use a deep learning method for HLA imputation [15]. On analysis, we identified 21 amino acid positions associated with classical T1D (DRß6, DRß13, DRß21, DRß31, DRß33, DRß39, DRß55, DRß68, DRß77, DQß15, DQß22, DQß29, DQß30, DQß31, DQß32, DQß34, DQß35, DQß61, DQß70, DQß75, and DQß85; *p* = 2.4 × 10^−10^, 3.4 × 10^−4^, 3.6 × 10^−11^, 6.8 × 10^−8^, 7.9 × 10^−7^, 7.9 × 10^−7^, 0.0268, 1.0 × 10^−8^, 1.0 × 10^−8^, 1.6 × 10^−8^, 0.0189, 4.7 × 10^−5^, 4.7 × 10^−5^, 2.9 × 10^−14^, 3.7 × 10^−9^, 4.7 × 10^−5^, 4.7 × 10^−5^, 0.0170, 1.2 × 10^−4^, 4.2 × 10^−6^, and 4.6 × 10^−5^, respectively), and 17 amino acid positions associated with LADA (DRß6, DRß13, DRß21, DRß31, DRß33, DRß39, DRß68, DRß77, DQß15, DQß29, DQß30, DQß31, DQß32, DQß34, DQß35, DQß70, and DQß85; *p* = 0.0317, 0.0012, 1.8 × 10^−4^, 0.0110, 2.3 × 10^−4^, 2.3 × 10^−4^, 0.0025, 0.0025, 0.0020, 5.8 × 10^−4^, 5.8 × 10^−4^, 6.6 × 10^−4^, 0.0012, 5.8 × 10^−4^, 5.8 × 10^−4^, 0.0020, and 2.9 × 10^−4^, respectively). Of these residues, the amino acids at HLA-DRß1 positions 13, 31, and 33 and at HLA-DQß1 positions 30, 70, 75, and 85 had variants in the HLA-DRB1 and -DQB1 alleles that we examined in this study. Thus, we compared the frequencies of the amino acids at HLA-DRß1 positions 13, 31, and 33 and at HLA-DQß1 positions 30, 70, 75, and 85 between classical T1D and controls, and at HLA-DRß1 positions 13, 31, and 33 and at HLA-DQß1 positions 30, 70, and 85 between LADA and controls.

The HLA-DRB1 and -DQB1 amino acid variants at DR and DQ positions that showed a strong association with classical T1D and LADA are presented in Table 2. Table 3 shows HLA-DRB1 amino acid variants at positions 13, 31, and 33, and HLA-DQB1 amino acid variants at positions 70 and 85, which confer susceptibility to or protection against classical T1D or LADA. DRß-Phe-31, DRß-Asn-33, and DQß-Val-85, which confer protection against classical T1D, are encoded by alleles which were found to confer susceptibility to or protection against the disease in the present study. Classical T1D-susceptible DQß-Leu-85 and LADA-protective DRß-Phe-31 are encoded by alleles which were found to confer susceptibility to or protection against the diseases in the present study. These amino acid variants might not be involved in the onset of the diseases. As a result, HLA-DRß1 position 13 and HLA-DQß1 positions 70 and 85 could be critical amino acid positions in the development of classical T1D and LADA.

Hu et al. demonstrated that conditioning on HLA-DQβ1 position 57, the second independent association with classical T1D was at HLA-DRß1 position 13 in Caucasian populations [16]. At this position, His and Ser conferred the strongest risk, whereas Arg and Tyr were protective. DRß-Ser-13 and DRß-Tyr-13 are respectively encoded by the HLA-DRB1*03:01 and -DRB1*07:01 alleles, which are rare in the Japanese population [2,9]. On the other hand, the HLA-DRB1*09:01 allele, which encodes Phe at HLA-DRß1 position 13 (Table 3), confer weak susceptibility to classical T1D in Caucasian populations [6]. Gerasimou et al. recently demonstrated that DQß-Arg-70 and DQß-Gly-70, respectively, confer susceptibility to and protection against classical T1D in Caucasian populations [17]. DQß-Arg-70 is encoded by the HLA-DQB1*02:01 allele, which is rare in Japanese populations [2,9]. DQß-Glu-70 is encoded by the HLA-DQB1*04:01 allele (Table 3), which is rare in Caucasian populations [6].

To the best of our knowledge, there is no report investigating the association of LADA with the amino acid variants in HLA-DRB1 and -DQB1 allotypes. The existence of DQα-Arg-52 and the absence of DQβ-Asp-57 did not confer susceptibility to LADA, as shown in classical T1D [18]. In the present study, DRß-Phe-13 encoded by the HLA-DRB1*09:01 allele and DRß-Arg-13 encoded by the HLA-DRB1*15:01 allele, respectively, confer susceptibility to and protection against LADA. Previous studies demonstrated that the HLA-DRB1*09 allele confers susceptibility to LADA in Asians [2,11,18,19], but not Caucasians [19,20]. The HLA-DRB1*15 allele confer protection against LADA both in Asians [2,11] and in Caucasians [20]. Although DRß-Ser-13 is not encoded by alleles, which confer susceptibility to and protection against LADA in the present study, it confers protection against LADA. DRß-Ser-13 is encoded by the HLA-DRB1*03:01 allele, which confers susceptibility to LADA in Caucasians [19,20] and in Asians other than the Japanese [18,19]. DRß-Ser-13 is also encoded by the HLA-DRB1*11:01 and -DRB1*14 alleles, which were found to have a protective trend against LADA in the present study and the previous study [19]. Desai et al. demonstrated that the HLA-DRB1*11:01 allele confers protection against LADA in Caucasians [20].

In the present study, DQß-Arg-70 and DQß-Leu-85 encoded by HLA-DQB1*03:02 and -DQB1*03:03 alleles, respectively, confer susceptibility to LADA. Previous reports demonstrated that the HLA-DQB1*03:02 allele, which constitutes the HLA-DRB1*04-DQA1*03:01-DQB1*03:02 haplotype in Caucasian populations, confers susceptibility to LADA, as well as to classical T1D [20,21,22,23,24]. On the other hand, the effect of the HLA-DQB1*03:03 allele on LADA is controversial. Previous reports demonstrated that this allele confers susceptibility to LADA in Asians [4,25]. However, another report demonstrated that this allele confers protection against LADA in Caucasian populations [19]. The HLA-DQB1*03:03 allele constitutes the HLA-DRB1*09-DQB1*03:03 haplotype. As with the HLA-DRB1*09:01 allele, the effect of the HLA-DQB1*03:03 allele on LADA depends on the ethnic group. Previous reports demonstrated that the HLA-DQB1*06:02 allele, which encodes DQß-Gly-70 and DQß-Val-85, confers protection against LADA [20,24,26]. However, the protective effect of this allele on LADA was not as strong as that on classical T1D [26,27]. Taken together, the protective effect of DQß-Gly-70 and DQß-Val-85 on LADA might be limited.

There are several limitations in this study. First, we were unable to demonstrate the association of DQβ-Asp-57 with classical T1D. Although the HLA-DQB1*0601 and -DQB1*06:02 alleles, which encode DQβ-Asp-57, were found to confer protection against classical T1D in the present study, a blind association analysis did not detect the association of HLA-DRβ1 position 57 with classical T1D. However, previous studies demonstrated that DQβ-Asp-57 did not confer protection against classical T1D, neither in Asians [28,29,30,31,32,33] nor in Caucasians [34,35]. In addition, we did not analyze *HLA-DQA1* alleles, *HLA-DP* loci, or HLA class I alleles. Recently, Xia et al. demonstrated that HLA-DP loci are associated with classical T1D [36]. Mishra et al. demonstrated that the HLA class I association may be a genetic discriminator between LADA and classical T1D [37]. We could not exclude the primary role of these loci in the development of classical T1D and LADA.

## 4. Conclusions

HLA-DRβ1 position 13 and HLA-DQß1 position 70 could be critical amino acid positions in the development of classical T1D and LADA. DRß-Phe-13 confers susceptibility to classical T1D and LADA, and DRß-Arg-13 and DQß-Gly-70 confer protection against the diseases. In addition, DRß-His-13 and DQß-Glu-70 confer susceptibility to classical T1D, and DRß-Ser-13 and DQß-Arg-70 confer protection against and susceptibility to LADA, respectively. Such novel alleles could guide autoantigen discovery and tolerance immunotherapies. Further studies are required to determine the underlying mechanisms of these differences. 

## Figures and Tables

**Table 1:** HLA-DRB1 and -DRB1 allele frequencies in patients with classical T1D, patients with LADA, and in controls.

Allele	Allele Frequency (Number)			Classical T1D vs. Control			LADA vs. Control		
	Classical T1D (*n* = 144)	LADA (*n* = 204)	Control (*n* = 516)	*p*	*Pc*	OR (95%CI)	*p*	*Pc*	OR (95%CI)
DRB1*01:01	4.9 (7)	4.9 (10)	3.9 (20)	0.37	—	1.27 (0.53–3.06)	0.33	—	1.28 (0.59–2.78)
DRB1*04:01	0.7 (1)	2.0 (4)	1.4 (7)	0.45	—	0.51 (0.06–4.17)	0.38	—	1.45 (0.42–5.02)
DRB1*04:03	0.7 (1)	3.4 (7)	3.9 (20)	0.037	—	0.17 (0.02–1.30)	0.49	—	0.88 (0.37–2.12)
DRB1*04:05	29.2 (42)	16.2 (33)	13.2 (68)	1.2 × 10^−5^	6.0 × 10^−5^	**2.71** (1.75–4.22)	0.18	—	1.27 (0.81–2.00)
DRB1*04:06	0.7 (1)	4.4 (9)	3.5 (18)	0.056	—	0.19 (0.03–1.46)	0.35	—	1.28 (0.56–2.89)
DRB1*04:07	0.7 (1)	2.9 (6)	0.4 (2)	0.52	—	1.80 (0.16–20.0)	0.0080	0.0400	**7.79** (1.56–38.9)
DRB1*04:10	2.1 (3)	3.0 (5)	2.1 (11)	0.64	—	0.98 (0.27–3.55)	0.10	—	0.23 (0.03–1.76)
DRB1*08:02	9.0 (13)	6.9 (14)	3.5 (18)	0.0080	0.0183	**2.75** (1.31–5.75)	0.041	0.15	2.04 (0.99–4.18)
DRB1*08:03	2.1 (3)	4.4 (9)	6.4 (33)	0.027	—	0.31 (0.09–1.03)	0.20	—	0.68 (0.32–1.44)
DRB1*09:01	27.1 (39)	25.0 (51)	13.8 (71)	2.2 × 10^−4^	7.2 × 10^−4^	**2.33** (1.49–3.63)	3.1 × 10^−4^	0.0031	**2.09** (1.40–3.13)
DRB1*11:01	0.0 (0)	1.5 (3)	3.7 (19)	0.0086	0.0185	**0**	0.089	—	0.39 (0.11–1.33)
DRB1*12:01	0.7 (1)	3.4 (7)	2.5 (13)	0.15	—	0.27 (0.04–2.09)	0.33	—	1.38 (0.54–3.50)
DRB1*12:02	0.7 (1)	0.5 (1)	2.1 (11)	0.22	—	0.32 (0.04–2.51)	0.10	—	0.23 (0.03–1.76)
DRB1*13:02	18.1 (26)	6.4 (13)	5.6 (29)	1.0 × 10^−5^	7.5 × 10^−5^	**3.70** (2.10–6.52)	0.41	—	1.14 (0.58–2.25)
DRB1*14:05	0.0 (0)	1.0 (2)	3.1 (16)	0.0185	0.0370	**0**	0.077	—	0.31 (0.07–1.36)
DRB1*14:06	0.7 (1)	0.5 (1)	2.1 (11)	0.22	—	0.32 (0.04–2.51)	0.10	—	0.23 (0.03–1.76)
DRB1*14:54	0.7 (1)	2.5 (5)	4.3 (22)	0.024	—	0.16 (0.02–1.18)	0.18	—	0.56 (0.21–1.51)
DRB1*15:01	0.0 (0)	3.9 (8)	11.6 (60)	1.7 × 10^−7^	5.2 × 10^−6^	**0**	5.8 × 10^−4^	0.0043	**0.31** (0.15–0.66)
DRB1*15:02	0.7 (1)	7.4 (15)	8.9 (46)	9.0 × 10^−5^	3.4 × 10^−4^	**0.07** (0.01–0.52)	0.30	—	0.81 (0.44–1.45)
DQB1*03:01	2.8 (4)	6.4 (13)	12.0 (62)	2.9 × 10^−4^	8.7 × 10^−4^	**0.21** (0.08–0.59)	0.0152	0.06	0.50 (0.27–0.93)
DQB1*03:02	10.4 (15)	17.6 (36)	10.1 (52)	0.51	—	1.04 (0.57–1.90)	0.0046	0.0278	**1.91** (1.21–3.03)
DQB1*03:03	26.4 (38)	26.0 (53)	14.5 (75)	0.0010	0.0024	**2.11** (1.35–3.29)	3.1 × 10^−4^	0.0046	**2.06** (1.39–3.07)
DQB1*04:01	29.2 (42)	16.2 (33)	13.0 (67)	9.0 × 10^−6^	9.0 × 10^−5^	**2.76** (1.77–4.29)	0.16	—	1.29 (0.82–2.03)
DQB1*04:02	4.2 (6)	2.5 (5)	3.9 (20)	0.52	—	1.08 (0.43–2.74)	0.24	—	0.62 (0.23–1.68)
DQB1*05:01	4.9 (7)	4.9 (10)	4.5 (23)	0.49	—	1.10 (0.46–2.61)	0.47	—	1.11 (0.52–2.36)
DQB1*05:02	0.7 (1)	1.5 (3)	3.3 (17)	0.069	—	0.21 (0.03–1.56)	0.14	—	0.44 (0.13–1.51)
DQB1*05:03	0.0 (0)	2.5 (5)	5.4 (28)	8.6 × 10^−4^	0.0024	**0**	0.06	—	0.44 (0.17–1.15)
DQB1*06:01	2.8 (4)	11.8 (24)	14.9 (77)	1.1 × 10^−5^	6.6 × 10^−5^	**0.16** (0.06–0.45)	0.16	—	0.76 (0.47–1.24)
DQB1*06:02	0.0 (0)	3.4 (7)	11.6 (60)	1.7 × 10^−7^	5.2 × 10^−6^	**0**	2.1 × 10^−4^	0.0064	**0.27** (0.12–0.60)
DQB1*06:04	17.4 (25)	6.4 (13)	5.4 (28)	1.6 × 10^−5^	6.9 × 10^−5^	**3.66** (2.06–6.51)	0.37	—	1.19 (0.60–2.34)

T1D, type 1 diabetes; LADA, latent autoimmune diabetes in adults; OR, odds ratio. Significant ORs are expressed in bold.

**Table 2:** HLA-DRB1 and -DQB1 amino acid frequencies at DR and DQ positions that showed an association with T1D and LADA in patients with classical T1D, those with LADA, and in controls.

Amino Acid Position	Amino Acid Variants	Allele Frequency (Number)			Classical T1D vs. Control			LADA vs. Control		
		Classical T1D (*n* = 144)	LADA (*n* = 204)	Control (*n* = 516)	*p*	*Pc*	OR (95%CI)	*p*	*Pc*	OR (95%CI)
DRß13	Phe	31.9 (46)	29.9 (61)	18.2 (94)	4.2 × 10^−4^	0.0012	**2.11** (1.39–3.19)	5.4 × 10^−4^	0.0020	**1.92** (1.32–2.78)
	His	34.7 (50)	29.4 (60)	24.4 (126)	0.0099	0.0231	**1.65** (1.11–2.45)	0.10	—	1.29 (0.90–1.85)
	Tyr	0.7 (1)	0.0 (0)	0.8 (4)	0.70	—	0.90 (0.10–8.07)	0.26	—	0
	Gly	12.5 (18)	15.7 (32)	14.5 (75)	0.32	—	0.84 (0.48–1.46)	0.39	—	1.09 (0.70–1.72)
	Ser	19.4 (28)	13.7 (28)	20.7 (107)	0.42	—	0.92 (0.58–1.47)	0.0177	0.0374	**0.61** (0.39–0.96)
	Arg	0.7 (1)	11.3 (23)	21.3 (110)	2.8 × 10^−12^	6.0 × 10^−11^	**0.03** (0.004–0.19)	8.9 × 10^−4^	0.0024	**0.47** (0.29–0.76)
DRß31	Ile	31.9 (46)	29.9 (61)	176 (91)	2.3 × 10^−4^	7.9 × 10^−4^	**2.19** (1.45–3.33)	2.7 × 10^−4^	0.0013	**1.99** (1.37–2.90)
	Phe	68.1 (98)	70.1 (143)	81.8 (422)	4.2 × 10^−4^	0.0012	**0.48** (0.31–0.72)	5.4 × 10^−4^	0.0020	**0.52** (0.36–0.76)
	Val	0.0 (0)	0.0 (0)	0.6 (3)	0.48	—	0	0.37	—	0
DRß33	Asn	65.3 (94)	70.6 (144)	75.6 (390)	0.0099	0.0231	**0.61** (0.41–0.90)	0.10	—	0.78 (0.54–1.11)
	His	34.7 (50)	29.4 (60)	24.4 (126)	0.0099	0.0231	**1.65** (1.11–2.45)	0.10	—	1.29 (0.90–1.85)
DQß30	Ser	0.7 (1)	0.0 (0)	0.8 (4)	0.70	—	0.90 (0.10–8.07)	0.26	—	0
	Tyr	76.4 (110)	83.8 (171)	80.2 (414)	0.19	—	0.80 (0.51–1.24)	0.16	—	1.28 (0.83–1.97)
	His	22.9 (33)	16.2 (33)	19.0 (98)	0.18	—	1.27 (0.81–1.98)	0.22	—	0.82 (0.53–1.27)
DQß70	Arg	61.1 (88)	68.1 (139)	58.0 (299)	0.28	—	1.14 (0.78–1.66)	0.0070	0.0167	**1.55** (1.10–2.19)
	Glu	33.3 (48)	18.6 (38)	16.9 (87)	2.5 × 10^−5^	1.1 × 10^−4^	**2.47** (1.63–3.74)	0.32	—	1.13 (0.74–1.72)
	Gly	5.6 (8)	13.2 (27)	25.2 (130)	1.6 × 10^−8^	1.7 × 10^−7^	**0.18** (0.08–0.37)	2.2 × 10^−4^	0.0042	**0.45** (0.29–0.71)
DQß75	Val	39.6 (57)	27.5 (56)	30.8 (159)	0.0308	0.054	1.47 (1.00–2.16)	—	—	—
	Leu	60.4 (87)	72.5 (148)	69.2 (357)	0.0308	0.054	0.68 (0.46–1.00)	—	—	—
DQß85	Leu	73.6 (106)	68.6 (140)	54.3 (280)	1.7 × 10^−5^	1.2 × 10^−4^	**2.35** (1.56–3.54)	2.6 × 10^−4^	0.0025	**1.84** (1.31–2.60)
	Val	26.4 (38)	31.4 (64)	45.7 (236)	1.7 × 10^−5^	1.2 × 10^−4^	**0.43** (0.28–0.64)	2.6 × 10^−4^	0.0025	**0.54** (0.39–0.76)

T1D, type 1 diabetes; LADA, latent autoimmune diabetes in adults; OR, odds ratio. Significant ORs are expressed in bold.

**Table 3:** HLA-DRB1 amino acid variants at positions 13, 31, and 33, and HLA-DQB1 amino acid variants at positions 70 and 85, which confer susceptibility to or protection against classical T1D or LADA.

Amino Acid Position	Amino Acid Variants	Classical T1D		LADA	
			Allotypes		Allotypes
DRß13	Phe	S	(S) DRB1*09:01	S	(S) DRB1*09:01
	His	S	(S) DRB1*04:05	—	
	Ser	—		P	None
	Arg	P	(P) DRB1*15:01, DRB1*15:02	P	(P) DRB1*15:01
DRß31	Ile	S	(S) DRB1*09:01	S	(S) DRB1*09:01
	Phe	P	(S) DRB1*04:05, DRB1*08:02, DRB1*13:02 (P) DRB1*11:01, DRB1*14:05, DRB1*15:01, DRB1*15:02	P	(S) DRB1*04:07 (P) DRB1*15:01
DRß33	Asn	P	(S) DRB1*08:02, DRB1*09:01, DRB1*13:02	—	
	His	S	(P) DRB1*11:01, DRB1*14:05, DRB1*15:01, DRB1*15:02	—	
DQß70	Arg	—		S	(S) DQB1*03:02, DQB1*03:03
	Glu	S	(S) DQB1*04:01	—	
	Gly	P	(P) DQB1*05:03, DQB1*06:02	P	(P) DQB1*06:02
DQß85	Leu	S	(S) DQB1*03:03, DQB1*04:01	S	(S) DQB1*03:02, DQB1*03:03
	Val	P	(P) DQB1*03:01	P	(P) DQB1*06:02

S or P, respectively, indicates susceptibility to or protection against the diseases based on HLA-DRB1 and -DQB1 allotypes or amino acids. T1D, type 1 diabetes; LADA, latent autoimmune diabetes in adults. Allotypes which confer susceptibility to or protection against T1D or LADA in this study.

## Data Availability

The data presented in this study are available on request from the corresponding author.

## Abbreviations

T1D	Type 1 diabetes
LADA	latent autoimmune diabetes in adults
HLA	human leukocyte antigen
GAD	glutamic acid decarboxylase
GAD-Ab	GAD antibody
IA-2Ab	insulinoma-associated antigen-2 antibody
SBT	sequence-based typing
BIGDAWG	Bridging ImmunoGenomic Data-Analysis Workflow Gaps
BMI	body mass index
